# Nightmare distress, insomnia and resilience of nursing staff in the post-pandemic era

**DOI:** 10.3934/publichealth.2024003

**Published:** 2023-12-18

**Authors:** Argyro Pachi, Athanasios Tselebis, Christos Sikaras, Eleni Paraskevi Sideri, Maria Ivanidou, Spyros Baras, Charalampos Milionis, Ioannis Ilias

**Affiliations:** 1 Psychiatric Department, Sotiria Thoracic Diseases Hospital of Athens, 11527 Athens, Greece; 2 Nursing Department, Sotiria Thoracic Diseases Hospital of Athens, 11527 Athens, Greece; 3 Emergency Department of General Hospital of Athens Korgialeneio-Benakeio Hellenic Red Cross, 11526, Athens, Greece; 4 Department of Endocrinology, “Elena Venizelou” Hospital, 11521 Athens, Greece

**Keywords:** nightmares, insomnia, resilience, nurses, COVID-19, mediation analysis

## Abstract

**Introduction:**

The pandemic has led to notable psychological challenges among healthcare professionals, including nurses.

**Objective:**

Our aims of this study were to assess insomnia and nightmare distress levels in nurses and investigate their association with mental resilience.

**Methods:**

Nurses participated in an online survey, which included the Nightmare Distress Questionnaire (NDQ), Brief Resilience Scale (BRS) and Athens Insomnia Scale (AIS). Demographic information, such as age, professional experience and gender, was also collected.

**Results:**

The study included 355 female and 78 male nurses. Findings revealed that 61.4% had abnormal AIS scores, 7% had abnormal NDQ scores and 25.4% had low BRS scores. Female nurses had higher AIS and NDQ scores but lower BRS scores compared to males. BRS demonstrated negative correlations with both AIS and NDQ. Multiple regression analysis indicated that NDQ accounted for 24% of the AIS variance, with an additional 6.5% explained by the BRS. BRS acted as a mediator, attenuating the impact of nightmares on insomnia, with gender moderating this relationship.

**Conclusions:**

Nursing staff experienced heightened sleep disturbances during the pandemic, with nightmares and insomnia being prevalent. Nightmares significantly contributed to insomnia, but mental resilience played a vital role in mitigating this effect. Strategies are warranted to address the pandemic's psychological impact on nursing professionals.

## 1. Introduction

In early May 2023, the World Health Organization (WHO) declared the conclusion of the global health emergency caused by the Coronavirus Disease 2019 (COVID-19) [1],[2]. COVID-19 was first reported in late 2019 and was officially characterized as a pandemic on March 11, 2020 [3]. The emergence of the COVID-19 pandemic marked an unprecedented global health crisis, setting it apart from previous infectious disease outbreaks and health emergencies [4]. Health crises have played a significant role in shaping history and have had a profound impact on human life and society [5]. From the Spanish flu to the HIV/AIDS epidemic the unique characteristics of the COVID-19 pandemic have presented distinct challenges [6]. The speed at which the COVID-19 virus spread globally was unparalleled. With modern transportation and interconnected economies, the virus traversed borders swiftly, creating a truly worldwide health crisis [7]. This rapid transmission posed challenges for containment and required nontraditional levels of international cooperation [8],[9]. Unlike some previous health crises where symptoms were often a clear indication of infection, carriers of the SARS-CoV-2 virus could unknowingly spread the disease [10]. This added a layer of complexity to tracking and controlling the virus's spread. Disparities in access to digital resources and the so-called digital divide became more pronounced, impacting the ability of certain populations to cope with the crisis [11]. The rapid development of COVID-19 vaccines was a testament to scientific advancements, but ensuring their fair distribution and overcoming logistical barriers posed additional issues [12]. Lockdowns, travel restrictions and disruptions in the supply chain had cascading effects on the global economy, distinguishing the COVID-19 crisis from previous health-related economic hurdles [13]. The pandemic also immensely increased the pressure on healthcare systems by raising the demand for certain treatments [14].

Throughout the pandemic, substantial psychological challenges emerged or intensified within the general populace [15],[16] and, notably, among healthcare professionals, with a particular emphasis on nursing staff [17]–[19]. Nurses constitute the largest professional contingent among healthcare providers and hold a pivotal role in the operation of healthcare systems, all while executing a profession demanding both physical and mental acumen [20]. Consequently, it is not surprising that researchers, both prior to and during the pandemic, expressed keen interest in exploring the physical and psychological factors influencing the health of nurses [21]. Among the facets under investigation in this regard, sleep-related issues garnered considerable attention, as evidenced by a multitude of publications [22]–[24].

Sleep constitutes a fundamental biological necessity for humans, playing a crucial role in maintaining overall health and ensuring safe working environments [24]. A standard night's sleep duration of seven to eight hours is linked to a decreased risk of physical ailments, including hypertension and diabetes, and is also associated with a reduced likelihood of workplace errors [23]. Among nursing staff, sleep disruptions are primarily linked to work-related factors such as extended work hours and shift schedules [25]. Stress, anxiety and depression contribute to sleep disturbances in both the general population and nursing professionals [24]. Additionally, nightmares are recognized as another influential factor contributing to sleep disorders [26]. Nightmares can lead to disturbed sleep patterns. When individuals experience frightening or emotionally distressing dreams, they may wake up abruptly, making it challenging to return back to sleep. Recurrent nightmares can create a fear of going to sleep, as individuals may be anxious about experiencing another disturbing dream. This fear can contribute to insomnia [27]. During the pandemic a recent research has revealed that frontline medical professionals suffered from frequent nightmares that adversely affected their sleep duration and efficiency [28].

Nightmare disorder is characterized by recurring and vivid distressing dreams that disrupt sleep, potentially causing daytime impairment. Its prevalence in the general population ranges from 2% to 5% [29],[30]. Among healthcare professionals, nurses stand out as a high-risk group [26]. Contributing factors to this elevated risk include irregular work schedules [31] and the inherently stressful healthcare environment [32]. However, a significant factor is nurses' exposure to trauma. Their frontline role in patient care frequently exposes them to traumatic events, such as witnessing severe injuries and fatalities, as well as experiencing vicarious trauma [32]. Furthermore, many researchers argue that pandemics, like the recent one, and the associated restrictive measures can induce trauma-related symptoms [33]–[35]. Studies report that the gravity and chronicity of the traumatic experience is usually associated with an elevated risk of nightmares [36],[37]. The pandemic experience triggered a significant increase in nightmares in the general population particularly in hospital workers where up to half of them suffered from nightmares during the pandemic [28],[38]. Confronting COVID-19 for healthcare workers is equivalent to trauma exposure and literature suggests that trauma-induced nightmares could last for a lifetime with devastating consequences [39]. Therefore, there is compelling evidence that nursing personnel may experience an amplified burden of nightmares as the pandemic subsides. It is essential to underscore that the consideration of nightmares is of paramount importance due to their association with various mental health conditions, most notably suicide risk [26]. The characteristics of trauma-related nightmares are influenced among other factors by an individual's psychological resiliency [40],[41].

Resilience, as defined by the American Psychological Association, denotes “the process of adapting effectively in the face of adversity, trauma, tragedy, threats or substantial stressors” [42]. It's noteworthy that resilience possesses two defining characteristics: its changeable and its position along a continuum, rather than existing in a binary framework of presence or absence [43]–[45]. Existing studies support an inverse correlation between resilience and insomnia [46],[47]. Research among nursing professionals has indicated that heightened resilience levels are associated with enhanced well-being and reduced psychological distress [48]. During the pandemic crisis, it was suggested that healthcare workers with elevated psychological resilience had an advantage in coping with pandemic-induced psychological stress and achieving successful recovery compared to those with lower resilience levels [49]. Recent research explored the role of resilience in the increase in nightmares among young adults during the COVID-19 pandemic [50]. Notably, there is a gap in the literature regarding the relationship between resilience and nightmares among nurses.

In this study, conducted two months after the official conclusion of the pandemic crisis as declared by the WHO, we aimed to evaluate nightmare distress manifested as a general distress, its impact on sleep and on daily reality perception and assess the severity of insomnia, namely; sleep onset, night and early-morning waking, sleep time, sleep quality, frequency and duration of complaints, distress caused by the experience of insomnia and interference with daily functioning, among nurses. Additionally, we sought to explore potential associations between insomnia, nightmare distress and mental resilience defined as the ability to bounce back or recover from stress. It was hypothesized that nightmare distress would be positively associated with insomnia and that mental resilience would mediate the effect of nightmare distress on insomnia.

Key research questions included:

1. What are the levels of nightmare distress, insomnia and resilience among nurses following the pandemic crisis?

2. Is there a correlation between nightmare distress, insomnia and resilience in this population?

3. Does resilience serve as a mediator in the relationship between nightmares and insomnia?

## 2. Participants and methods

This was a study involving Greek nurses. Data collection relied on self-report questionnaires distributed via email. The email invitation included an anonymous link granting access to the online survey platform on Google™ Forms. On the initial page of the online questionnaire, participants were presented with a consent form outlining the voluntary nature of their participation.

Participants' email addresses were sourced from scientific and professional directories of Greek nurses. The study sample comprised nurses who voluntarily responded to the email, forming a convenience sample. No specific measures were implemented to enhance response rates.

### 2.1. Ethical considerations

In the invitation, nurses were provided with comprehensive information about our purpose and design. We ensured electronic informed consent by including the initial question, “Do you agree to participate in this study?” in the online questionnaire. Only nurses responding affirmatively were permitted to proceed with completing the questionnaire. We adhered to ethical principles in accordance with the Declaration of Helsinki, guidelines established by the International Committee of Medical Journal Editors and compliance with the General Data Protection Regulation (GDPR–2016/679) of the European Union. Ethical approval was obtained from the Clinical Research Ethics Committee of “Sotiria” General Hospital (Approval Number: 20649/23).

### 2.2. Study participants

The study was conducted between June 10 and June 30, 2023. With a target population of 27103 nurses [19],[21],[24], at a confidence level of 95%, a margin of error of 5% and a percentage of our sample picking a particular answer of 50%, the adequate sample of nurses was set at 379 participants. Considering that the response rate in past studies [48],[49] was more than 60%, a total of 600 email invitations were dispatched, with 433 nurses consenting to participate, resulting in a response rate of 72.2%. In the first 7 days we had received over 80% of the responses.

The adequacy of the sample was confirmed using G-Power Version 3.1 software (G*Power Team, Heinrich-Heine-Universität, Düsseldorf, Germany) [51],[52]. With a sample size of 433, six coefficients and an alpha level of 0.05, the calculated statistical power was 1.00. Also, in Appendix we specify the formula for sample size determination. Furthermore, a Monte Carlo power analysis was conducted for an individual mediation model [52]. In this analysis, considering 433 subjects, 5000 replicates and a 99% confidence level, the calculated power was also 1.00.

### 2.3. Measurement tools

Upon obtaining consent and before completing the questionnaires, participants provided information regarding their gender, age and years of professional experience. Subsequently, the nurses proceeded to complete the following questionnaires, which have successfully been used in previous studies in Greek nurses:

-The Nightmare Distress Questionnaire (NDQ) [53]–[55]: This is the most widely employed questionnaire for evaluating the distress associated with nightmares. Comprising 13 questions, the NDQ encompasses a spectrum of issues linked to nightmares [34]. Responses to the questionnaire items are rated on a on a 5-point response set, with higher scores indicative of heightened distress levels. Most items like “Do you have difficulties coping with nightmares?” range from 1 = never to 5 = always, whereas items like “Do nightmares interfere with the quality of your sleep?” are rated from 1 = not at all to 5 = a great deal. The last item assessing the interest in nightmare therapy is coded as follows: 1 = not at all interested to 5 = extremely interested. The total score on this questionnaire ranges from 13 to 65, and scores exceeding 39 are considered abnormal. The NDQ has demonstrated commendable internal consistency (Cronbach's alpha) with values ranging from α = 0.83 to α = 0.88 [55]. In our study, Cronbach's alpha was found to be α = 0.91, indicating high internal consistency.

-The Brief Resilience Scale (BRS): The BRS is a concise self-assessment tool comprising six questions, designed to gauge an individual's capacity to surmount stress and adversity. Responses to the questionnaire items are rated on a five-point Likert scale, ranging from 1 (strongly disagree) to 5 (strongly agree). The cumulative score on this scale falls within the range of 6–30, where higher scores denote greater resilience. To obtain an individual's resilience score, the total scale score is divided by the number of questions they answered. Accordingly, scores exceeding 4.30 are indicative of high resilience, while values below 2.99 indicate low resilience [48],[56],[57]. Previous studies have established the scale's robust internal consistency, with Cronbach's alpha coefficients reaching α = 0.86 [28]. In our current study, the Cronbach's alpha coefficient was determined to be α = 0.84, signifying good internal consistency.

-The Athens Insomnia Scale (AIS): The AIS is a widely utilized self-assessment questionnaire designed to evaluate sleep difficulties, aligning with the criteria for insomnia set forth in the International Classification of Diseases, 10th Revision (ICD-10) [23],[24]. Comprising eight items, respondents rate their responses on a 4-point response set, ranging from 0 to 3. The cumulative scale score spans from 0 to 24, with a diagnostic threshold set at 6. A score equal to or greater than 6 is indicative of clinical insomnia [58],[59]. The scale's creators have reported a robust internal consistency, with a Cronbach's alpha coefficient of α = 0.89 [59]. In our current study, we observed a Cronbach's alpha coefficient of α = 0.85, signifying good internal consistency.

### 2.4. Statistical analysis

We initially conducted descriptive statistical analyses, expressing continuous variables as means with accompanying standard deviations. To assess the sample's representativeness, we compared it to a previous study sample in terms of gender, age, and years of professional experience using chi-squared (*χ²*) tests and *t*-tests. Gender-related differences in continuous variables were identified using *t*-tests. The strength and direction of associations between variables were determined through Pearson's correlation analysis. We constructed a linear regression model to explore whether correlated variables served as statistically significant predictors of insomnia. Regression assumptions (linearity, independence, homoscedasticity and normality) were examined by visual inspection of pairwise scatter plots to confirm linearity, P-P plots to check for normality, residuals scatter plot for homoscedasticity, the Durbin–Watson test for independence of the residuals and the Variance Inflation Factor analysis (VIF) to verify the absence of multicollinearity in the data. Employing Hayes SPSS Process Macro [60],[61], we performed a mediation analysis. In this analysis, the predictor variable was NDQ, the mediation variable was BRS and the outcome variable was AIS. IBM SPSS version 20 was the software utilized for all analyses (IBM Corp., Armonk, NY, USA). The significance level was set at 0.05 (two-tailed).

## 3. Results

The study encompassed 355 female nurses and 78 male nurses. It's noteworthy that there were no statistically significant differences in terms of years of professional experience, age and gender between the participants in this study and the entire nursing workforce in the country [62],[63]. The mean values and standard deviations for the variables are presented in Table 1. Concerning nightmare distress, 7% exhibited abnormal values (NDQ ≥ 40), while 25.4% displayed low resilience (BRS ≤ 2.99). A substantial 61.4% recorded abnormal scores on the insomnia scale (AIS ≥ 6). Notably, the mean insomnia score (7.26 ± 4.13) in this study was statistically higher than that observed in Greek nurses at the onset of the pandemic (5.98 ± 4.24, *N* = 150) [23], as indicated by a sample *t*-test (*p* < 0.01). Calculating Hedges' g between the initial study and the current one revealed a small effect size (g: 0.31). In our sample, female participants, when compared to their male counterparts, exhibited statistically higher scores on the NDQ and AIS scales (*t*-test *p* ≤ 0.01) and lower scores on the BRS (*t*-test *p* ≤ 0.05), as illustrated in Table 1.

We observed a positive correlation between NDQ and AIS (Pearson Correlations *p* < 0.01, Table 2). Conversely, BRS demonstrated negative correlations with both NDQ and AIS (Pearson Correlations *p* < 0.01, Table 2), suggesting an inverse relationship. Age exhibited a positive correlation with the BRS scale and negative correlations with the NDQ and AIS scales (Pearson Correlations *p* < 0.01, Table 2), reflecting significant age-related trends. Work experience displayed positive correlations with the BRS scale and negative correlations with the AIS (Pearson Correlations *p* < 0.01, Table 2), indicating notable associations between these variables.

**Table 1.** General characteristics of nurses and Nightmare /Insomnia/ Resilience, scores in relation to gender.

**Participants**	**Descriptive statistics**	**Age**	**Work experience (in years)**	**Nightmare Distress Questionnaire (NDQ)**	**Athens Insomnia Scale (AIS)**	**Brief Resilience Scale (BRS)**
**Male (*N* = 78)**	Mean	46.64**	20.12	20.21**	6.05**	3.60*
	Std. Deviation	10.63	11.68	6.50	3.98	0.78
**Female (*N* = 355)**	Mean	43.09**	17.82	23.59**	7.53**	3.35*
	Std. Deviation	10.90	11.98	9.41	4.13	0.78
**Total (*N* = 433)**	Mean	43.73	18.23	22.97	7.26	3.39
	Std. Deviation	10.92	11.92	9.04	4.13	0.78

Notes: *p < 0.05; **p < 0.01.

**Table 2.** Correlations among age, work experience, NDQ, AIS and BRS.

**Pearson Correlation (*N* = 433)**		**Age**	**Work experience (in years)**	**Nightmare Distress Questionnaire (NDQ)**	**Athens Insomnia Scale (AIS)**
**Work experience (in years)**	***r***	0.868**			
**Nightmare Distress Questionnaire (NDQ)**	***r***	-0.161**	-0.170**		
**Athens Insomnia Scale (AIS)**	***r***	-0.109*	-0.075	0.521**	
**Brief Resilience Scale (BRS)**	***r***	0.218**	0.189**	-0.385**	-0.432**

Note: *Pearson Correlations *p* ≤ 0.05, **Pearson Correlations *p* ≤ 0.01.

We diligently assessed the fulfillment of necessary assumptions for regression analysis. To confirm linearity in the relationships between the variable we provided the pairwise scatter plots in Appendix. The absence of multicollinearity was evaluated via Variance Inflation Factor (VIF) analysis, yielding a value of 1.174 (Table 3) for the variables that participated in the interpretation of the independent variable, while for the excluded variables, the VIF value ranged from 1.021 to 1.059. The Durbin-Watson test, with a value of 1.973, was employed to scrutinize residual independence (Table 3). To verify the assumption of normality, we conducted a visual inspection of the Predicted Probability (P-P) plots. To assess homoscedasticity, we performed a visual inspection of the scatter plot depicting regression standardized residuals and regression standardized predicted values.

**Table 3.** Stepwise multiple regression (only statistically significant variables are included).

**Dependent Variable: Athens Insomnia Scale**	*R* Square	*R* Square Change	*Beta*	*t*	*p*	VIF	Durbin-Watson
**Nightmare Distress Questionnaire (NDQ)**	0.240	0.240	0.377	8.560	0.001*	1.174	1.973
**Brief Resilience Scale (BRS)**	0.305	0.065	-0.279	-6.334	0.001*	1.174	

Notes: *Beta* = standardized regression coefficient; *Correlations are statistically significant at the *p* < 0.001 level.

**Figure 1.**
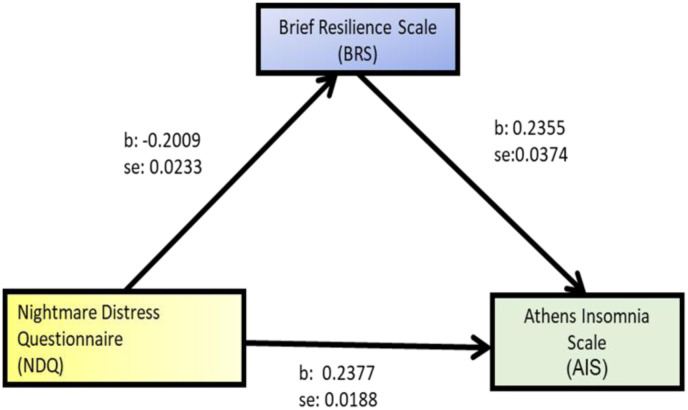
Mediation analysis of Brief Resilience Scale (BRS) on Nightmare Distress Questionnaire (NDQ)-Athens Insomnia Scale (AIS) relationship.

Employing the Stepwise method, we conducted a multiple regression analysis to pinpoint the key determinants of AIS scores. In this analysis, AIS served as the dependent variable, while the independent variables encompassed gender, age, work experience, NDQ and BRS. Our multiple regression analysis unveiled that the NDQ accounted for 24% of the variance, while the BRS contributed an additional 6.5% of the variance in AIS scores (Table 3). Notably, the remaining variables did not play a significant role in explaining the independent variable. We proceeded to investigate the hypothesis that the BRS acts as a mediator in the relationship between the NDQ and the AIS. To assess this mediation, we employed bootstrapping, using the Hayes SPSS Process Macro (Model 4). This entailed analyzing 5000 bootstrap samples to scrutinize whether BRS mediates the connection between NDQ and AIS. In this analysis, AIS served as the outcome variable, NDQ was the predictor variable and BRS functioned as the mediator variable (Table 4, Figure 1). Furthermore, work experience and age were introduced as covariates.

**Table 4.** Mediation analysis of Brief Resilience Scale (BRS) on Nightmare Distress Questionnaire (NDQ) - Athens Insomnia Scale (AIS) relationship.

Variable	*b*	*SE*	*t*	*p*	95% Confidence Interval	
					LLCI	ULCI
**NDQ→BRS**	-0.2009	0.0233	-8.6328	0.0001	-0.2466	-0.1551
**NDQ→AIS**	0.2377	0.0188	12.6441	0.0001	0.2008	0.2747
**NDQ→BRS→AIS**	-0.2355	0.0374	-6.2967	0.0001	-0.3091	-0.1620
Effects
Direct	0.1904	0.0195	9.7595	0.0001	0.1521	0.2288
Indirect*	0.0473	0.0099			0.0300	0.0686
Total	0.2377	0.0188	12.6441	0.0001	0.2008	0.2747

Note: 1) *Based on 5000 bootstrap samples. 2) Work experience and age were included in the analysis as covariates variables. They are not shown in the table as they did not give significant statistical results (*p* > 0.05).

Our mediation analysis revealed that the association between NDQ and AIS was indeed mediated by BRS, as is evident in Table 4 and Figure 1. Notably, the indirect effect of BRS was statistically significant [*B* = 0.0473, 95% *CI* (0.0300, 0.0686), *p* ≤ 0.01]. Additionally, even in the presence of the mediator BRS, the direct effect of NDQ on AIS remained significant [*b* = 0.1904, 95% *CI* (0.1521, 0.2288) *p* ≤ 0.001). The covariates, work experience and age did not exhibit statistically significant relationships in this context. In part, the BRS serves as a mediator in the relationship between the NDQ and the AIS. Furthermore, our model explains 20% of the variance in the outcome variable, AIS (Table 4).

Finally, we explored the moderating influence of gender in the association between NDQ and BRS. To conduct this moderation analysis, we employed the PROCESS method, model 7. Here, the NDQ functioned as the predictor variable, the AIS served as the outcome variable and the BRS acted as the mediator variable. Our analysis revealed that gender played a statistically significant moderating role in the relationship between the NDQ and the AIS through BRS, as elucidated in Table 5.

**Figure 2.**
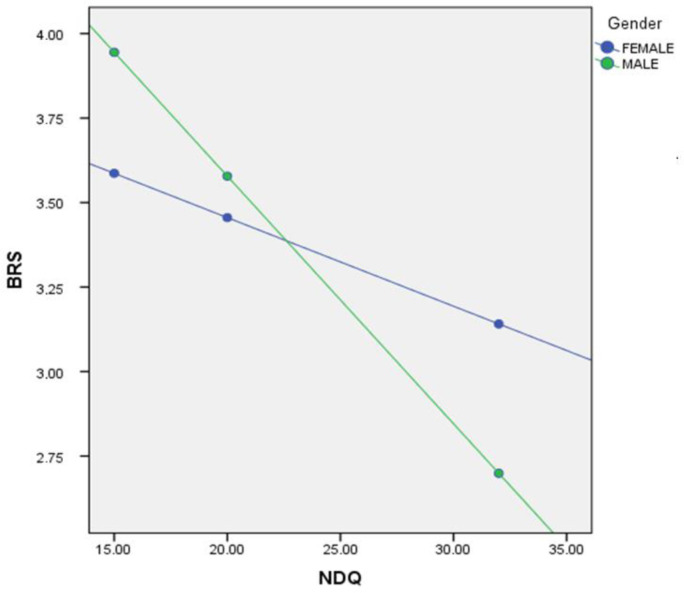
Moderated mediation analysis of the effect of Gender on the association between the Nightmare Distress Questionnaire (NDQ) and Athens Insomnia Scale (AIS) through Brief Resilience Scale (BRS).

In its role as a moderator, gender brings about alterations in the strength of the indirect effect observed in the aforementioned mediation analysis (Table 5, Figure 2). Notably, the index of moderated mediation was found to be statistically significant [*b* = -0.0645, 95% *CI* (-0.1100, -0.0284)]. This signifies compelling evidence for a moderated mediation effect. We observed distinct conditional indirect effects based on the gender moderator values. Specifically, the conditional indirect effect was more pronounced among males [*b* = 0.1045, 95% *CI* (0.0238, 0.00596)] in contrast to females [*b* = -0.0645 95% *CI* (-0.1100, -0.0284)].

**Table 5.** Moderated mediation analysis of the effect of Gender on the association between the Nightmare Distress Questionnaire (NDQ) and Athens Insomnia Scale (AIS) through Brief Resilience Scale (BRS).

**Outcome Variable: Brief Resilience Scale (BRS)**	***b***	***SE***	***t***	***p***
**Constant**	36.9778 [30.5778, 43.3779]	3.2591	11.3564	0.001
**Nightmare Distress Questionnaire (NDQ)**	-0.7174 [-1.0175, -0.4173]	0.1527	-4.6989	0.001
**GENDER**	-6.4232 [-9.7921, -3.0543]	1.7140	-3.7476	0.01
**Interaction (NDQ × GENDER)**	−0.2737 [0.1180, 0.4294]	0.0792	3.4558	0.01
**Direct effect of Nightmare Distress Questionnaire (NDQ) on Athens Insomnia Scale (AIS)**				
**Indirect effect: NDQ→BRS→AIS**	0.1904 [0.1521 ,0.2288]	0.0195	9.7595	0001
**MALES**	0.1045 [0.0620,0.1552]	0.0239		
**FEMALES**	0.0400 [0.0238, 0.00596]	0.0091		
**Index of Moderated Mediation**	-0.0645 [-0.1100, -0.0284]	0.0205		

## 5. Discussion

Even before the pandemic crisis, healthcare workers, including nurses, were known to be susceptible to the development of mental and psychological challenges [64]–[66]. These vulnerabilities became particularly pronounced during the peak of the pandemic crisis [16]. Considerable data have reported higher prevalence rates of anxiety, stress, depression, fatigue and post-traumatic stress disorder in nurses during this crisis [19],[21],[23]. Thus, the sleep disorders experienced by nurses today are likely to reflect the overall increase in psychological problems among nurses as a result of the extremely high pressure they experienced during the pandemic crisis. In the first year of the pandemic, insomnia was a major problem for 49.7% of nurses [23] a figure that jumped to 61.4% in the second year [24] same as we see today, which remains stable despite the end of the pandemic crisis.

In this study, we highlight a notable prevalence of nightmare distress among nursing staff. Numerous investigations conducted during the pandemic, particularly during the lockdown phases, have supported an increase in nightmares within the general population [67]–[69]. However, with the conclusion of lockdown measures, negative dream experiences have demonstrated a reduction [70]. Evidence supports that nightmares may serve an adaptive role against stressful situations and in some cases possibly facilitating traumatic memory extinction [71],[72]. Since the relationship between overactivation of the stress system and nightmares has been confirmed [73],[74], it is possible that while in the general population, we had acute overactivation of the stress system, during the pandemic, nurses may be grappling with a chronic form of overactivation. This rather unfavorable hypothesis suggests that despite the conventional conclusion of the pandemic crisis, nurses persist in encountering stress, whether in the form of post-traumatic stress or mental stress stemming from the deteriorating working conditions in the post-COVID era.

In nightmare aetiology, both trait factors such as neurotisism, ego strength or sensory processing sensitivity and state factors regarding the impact of distress on one's ability to efficiently cope with negative emotions are implicated [75]–[77]. This is consistent with the results from a research study demonstrating that current stress levels mediate the effect of neuroticism on nightmare frequency [75]. However, chronic nightmares are assumed to generate via the interplay of an increased hyperarousal and a fear extinction deficit [78]. Emotional dysregulation drives repetitive activation and recurrent processing of fear memories and consequently produce nightmares [72]. Involved neural networks are hyperactivated limbic structures and hypoactivated prefrontal areas. When the emotional load or stress level rises, the frequency of reactivation of fear memories and thereupon the occurrence of nightmares also increases [79]. Increased hyperarousal is argued as a key pathophysiological mechanism in insomnia disorder and studies reveal that patients diagnosed with this disorder manifest more frequent microarousals during REM sleep compared to people with good sleep [80]–[82]. This hyperarousal presenting as excessive beta activity during sleep amplifies information processing, thus supporting the aetiology of nightmare disorder in insomniacs [83].

Our study reaffirms a significant positive correlation between nightmares and insomnia. Specifically, it suggests that nightmares can elucidate 24% of the variance in insomnia. However, it is plausible that a cyclical dynamic exists, where nightmares trigger insomnia, and subsequently, insomnia exacerbates mental health issues while further increasing the prevalence of nightmares. The hyperactivity of the sympathetic nervous system or the hypothalamic–pituitary–adrenal axis (HPA) seems to generate a negative loop with sleep fragmentation and nightmares [84]. Nevertheless, our primary focus should center on identifying positive factors capable of disrupting this cycle. In this context, our study posits mental resilience as one such influential factor.

Our findings lend support to the notion of an inverse relationship between resilience and both insomnia and nightmares. Notably, resilience seems to exert a protective influence on sleep quality, effectively acting as a negative mediator in the connection between nightmares and insomnia. Research involving healthcare workers during the pandemic has consistently highlighted resilience as a safeguard against various mental health conditions, encompassing anxiety, depression, post-traumatic stress and sleep disturbances [85]–[87]. While there are other protective intrinsic factors such as sense of coherence [88],[89] or even extrinsic ones such as family support [90],[91] what makes a person resilient is the mixture of intrinsic and extrinsic characteristics [92],[93]. Therefore, interventions aimed at bolstering resilience should consider this holistic perspective, incorporating elements from both dimensions. In conclusion, when devising interventions to enhance the mental well-being of nurses and healthcare personnel, it's crucial to recognize the significance of organizational, social, personal and psychological factors. All these elements can play pivotal roles in promoting resilience and, consequently, mental health [94]. On the other hand, we emphasize that there is no strong evidence on which interventions best supported health worker resilience during the pandemic [95].

In this study, we underscore a gender disparity in sleep disturbances, with female nurses experiencing higher rates compared to their male counterparts. This observation aligns with recent analyses, revealing a significantly elevated prevalence of insomnia among women when compared to men [96]. Additionally, another study has identified female gender as an independent predictor of insomnia symptoms [97]. One plausible mechanism contributing to the increased prevalence of insomnia in women may involve the influence of progesterone and estrogen [98]. Sleep-related complaints tend to intensify during hormonal fluctuations associated with events such as menopause, puberty, menstrual cycles and pregnancy [99],[100]. Notably, menopausal women have shown improvements in sleep disturbances with estrogen therapy [100]. Further research indicates that adult women are more likely to report nightmares than men, although this distinction doesn't manifest in children or the elderly [101],[102]. Moreover, the heightened occurrence of anxiety and depression among females, coupled with the established link between sleep disorders and these mental health conditions, offers an additional explanatory factor [64],[102].

Mounting evidence argue on the topic of gender-specific differences and the complex relationships between desynchronization of biological rhythms and individual circadian preference [103]. Studies on female nurses identified genomic variants and evidenced hormonal phase desynchronization between nightshift and dayshift participants [104]. Results from an extensive study showed that nurses working shifts had more than 1.5-fold increased risk of nightmares, compared to nurses working daytime only and evidenced a strong correlation between nightmares and evening individual circadian preference (chronotype) [32]. Individual circadian preference affects the neurophysiological substrate of emotional processing activated during sleep and dreaming and this could explain the different occurrence of nightmares for women of the evening chronotype.

Gender assumes a distinctive role by serving as a moderator in the interplay between resilience and nightmares, particularly within the context where resilience functions as a mediator in the relationship between nightmares and insomnia. Notably, preceding and during the pandemic crisis, studies consistently demonstrate lower BRS scores in females compared to males [105],[106],[107]. Within the nursing community during the pandemic, gender has also emerged as a determinant of resilience, with females exhibiting lower resilience values than their male counterparts [108]. This heightened vulnerability is predominantly attributed to cultural factors, with women often navigating the delicate balance between professional responsibilities and increased family demands [108].

In relation to age and resilience, our findings reveal a positive correlation between older age and higher resilience. While the underlying reasons for this association remain less explored [109], it is postulated that older individuals may invest more time and effort in their health and well-being, but more plausible explanation seems to be that their accumulated life experiences may equip them with enhanced coping strategies in the face of adversity [110]. Also, studies evidence an age dependent decrease in nightmare frequency, but findings in this area are contradictory [79].

For decades, the nursing profession has faced many occupational shortcomings and pressures [111]. The emergence of COVID-19 has rendered these issues more apparent. The mental and physical fatigue of nurses and especially the lack of interventions inevitably lead to the resignation of professionals from their work [112],[113], putting the quality and safety of patients' care at serious risk. Also, it is worth mentioning that according to studies male nurses have a lower perceived sense of professional identity and higher attrition rates compared to female nurses and perhaps this is one of the reasons that those who remain are probably more resilient. As a consequence, nursing administrators struggle to persuade male nurses to remain in the nursing profession [114],[115].

As the pandemic crisis draws to a close, it becomes paramount to implement comprehensive strategies and interventions aimed at directly addressing its lingering psychological impact on nurses. These interventions should operate at individual, community and organizational levels, with a special focus on preventing long-term repercussions on nurses' mental health. Of particular concern are female nurses, who constitute the majority of nursing staff and appear to be more vulnerable than their male counterparts.

Finally, we would like to draw attention to the fact that psychological factors clearly cannot and certainly should not mask the existing deficits of a health care system. The conceptual approach to resilience finds practical application in explaining why there were nurses who did not experience insomnia and nightmares even though they were under pressure. The health system has the responsibility to compensate for this pressure by ensuring that staff benefit from proper working conditions, an optimal distribution of patients to nurses and a work timetable that guarantees adequate relaxation.

## 6. Methodological issues and Limitations of this study

Before concluding the discussion, several methodological issues have to be considered. The use of the nightmare distress instrument is more sensitive to detecting mental health issues, than is nightmare frequency alone. Stress or psychopathology is related to dream recall frequency but apart from insomnia we have not evaluated other mental health problems, personal life stressors, or other work-related factors. Also, we did not examine nightmare content and there are studies reporting on differences between women's pandemic dreams compared to men's, suggesting possible gender differences in the processing of threat or risk stimuli along with different adaptive behavioural responses to these cues in order to achieve survival [69]. Using retrospective questionnaires there might be possible underestimation of nightmare frequency, but the response rate might be related to having nightmares. Generally, nightmares are rarely reported to healthcare providers [116], perhaps due to lack of knowledge about treatment availability, but women tend to report nightmares more often than men, introducing social desirability as a source of bias. This is in conjunction with gender disproportionality, which might have skewed our results. Besides, the practical significance of the results can only be assessed in experimental and/or real-life conditions. Moreover, since there are potent treatment strategies for coping with insomnia and nightmares with stable long-term effects, it would be interesting to conduct longitudinal intervention studies to investigate rates of insomnia, nightmare distress and resilience as to current stressors. As to the gendered aspect of resilience, most studies support that males scored higher resilience levels compared to females, apart from reasons of heritability [117],[118]. Gender-specific differences regarding resilience levels were not observed in our previous study on nursing staff during the pandemic [48]. In addition, since results from other studies have been equivocal, gender has been identified as an inconclusive and unreliable factor in predicting resilience. This is likely due to the fact that resilience measurement tools are not gender specific [119]. In the present study the gendered aspect could be examined as to the finding that male nurses seem to receive more protection from psychological resilience as to nightmare distress.

Several limitations also warrant consideration. First, as already mentioned, the utilization of self-administered questionnaires introduced a subjective dimension to the assessment of the variables. Second, the exclusion of nurses without convenient internet access, given that invitations were exclusively distributed via email, may have resulted in a sample that does not comprehensively represent all nursing populations. Third, the observed gender disproportionality within the nursing samples raises potential concerns prohibiting the generalizability of findings to broader populations. Finally, due to the frequent rotation of the nursing staff, factors relating to the unit where the nurses worked, shifts at work department or even staff shortages were not included in the data collection.

## 7. Conclusions

Following the conclusion of the pandemic crisis, nursing staff have exhibited notably heightened levels of sleep disturbances. Moreover, nurses have reported elevated rates of nightmare distress compared to the general population, and insomnia has emerged as a prevalent issue among them. Nightmare distress has been identified as significant contributor to insomnia, with resilience serving as a protective mediator, mitigating the adverse impact of nightmare distress on sleep patterns. Gender has demonstrated a moderating influence in the interplay between nightmares and resilience, with women experiencing relatively increased nightmare distress and lower levels of resilience and men receiving more protection from psychological resilience as to nightmare distress. As we move forward, it is of paramount importance to implement targeted strategies and interventions to effectively address the pandemic's psychological repercussions on nurses.

## Use of AI tools declaration

The authors declare they have not used artificial intelligence (AI) tools in the creation of this article.


