# Supplementary material for: Nightmare distress, insomnia and resilience of nursing staff in the post-pandemic era
Source: AIMS Public Health. 2023 Dec 18;11(1):36–57. doi: 10.3934/publichealth.2024003 (PMC11007420; doi:10.3934/publichealth.2024003)
Supplement: Supplementary file 1 [file publichealth-11-01-003-s001.pdf]

AIMS Public Health

AIMS Public Health, 11(1): 36–57.
DOI: 10.3934/publichealth.2024003
Received: 04 October 2023
Revised: 10 December 2023
Accepted: 11 December 2023
Published: 18 December 2023

http://www.aimspress.com/journal/aimsph

*Research article*

# Nightmare distress, insomnia and resilience of nursing staff in the post-pandemic era

**Argyro Pachi[1], Athanasios Tselebis[1,*], Christos Sikaras[2], Eleni Paraskevi Sideri[3], Maria Ivanidou[1], Spyros Baras[1], Charalampos Milionis[4] and Ioannis Ilias[4]**

[1] Psychiatric Department, Sotiria Thoracic Diseases Hospital of Athens, 11527 Athens, Greece
[2] Nursing Department, Sotiria Thoracic Diseases Hospital of Athens, 11527 Athens, Greece
[3] Emergency Department of General Hospital of Athens Korgialeneio-Benakeio Hellenic Red Cross, 11526, Athens, Greece
[4] Department of Endocrinology, "Elena Venizelou" Hospital, 11521 Athens, Greece

* **Correspondence:** Email: atselebis@yahoo.gr; Tel: +302107763186.

## Supplementary I

Formula for determining sample size:

$$Sample\ Size = \frac{\frac{z^2xp(1-P)}{e^2}}{1+\frac{z^2xp(1-P)}{e^2N}} \tag{1}$$

Note: **z** is the z score = 1.96 (Confidence Level 0.95); **e** is the margin of error = 0.05; **N** is the population size = 27105; **P** is the population proportion = 0.5.

# Supplementary II

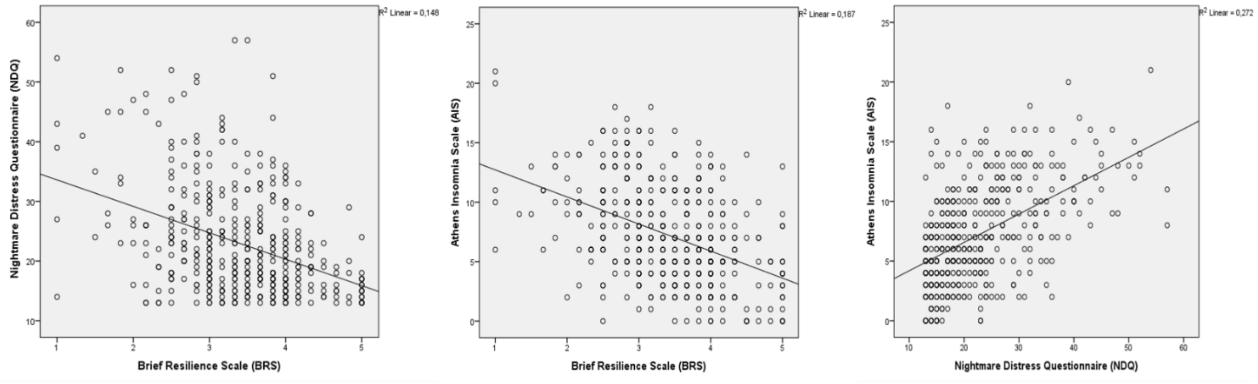


**Figure S1.** Pairwise scatter plots of Nightmare Distress Questionnaire (NDQ), Brief Resilience Scale (BRS), and Athens Insomnia Scale (AIS).

AIMS Press

© 2024 the Author(s), licensee AIMS Press. This is an open access article distributed under the terms of the Creative Commons Attribution License (http://creativecommons.org/licenses/by/4.0)
